# Pathophysiology and biomarkers of acute respiratory distress syndrome

**DOI:** 10.1186/2052-0492-2-32

**Published:** 2014-05-07

**Authors:** Seitaro Fujishima

**Affiliations:** Center for General Internal Medicine and Education, School of Medicine, Keio University, Tokyo, 160-8582 Japan

**Keywords:** Berlin definition, Cytokine, IL-8, IL-18, Leptin, Damage-associated molecular patterns

## Abstract

Acute respiratory distress syndrome (ARDS) is defined as an acute-onset, progressive, hypoxic condition with radiographic bilateral lung infiltration, which develops after several diseases or injuries, and is not derived from hydrostatic pulmonary edema. One specific pathological finding of ARDS is diffuse alveolar damage. In 2012, in an effort to increase diagnostic specificity, a revised definition of ARDS was published in *JAMA*. However, no new parameters or biomarkers were adopted by the revised definition. Discriminating between ARDS and other similar diseases is critically important; however, only a few biomarkers are currently available for diagnostic purposes. Furthermore, predicting the severity, response to therapy, or outcome of the illness is also important for developing treatment strategies for each patient. However, the PaO_2_/FIO_2_ ratio is currently the sole clinical parameter used for this purpose. In parallel with progress in understanding the pathophysiology of ARDS, various humoral factors induced by inflammation and molecules derived from activated cells or injured tissues have been shown as potential biomarkers that may be applied in clinical practice. In this review, the current understanding of the basic pathophysiology of ARDS and associated candidate biomarkers will be discussed.

## Introduction

Acute respiratory distress syndrome (ARDS) is defined as an acute-onset, progressive, hypoxic condition characterized by bilateral lung infiltration on chest X-ray or computed tomography [1]. ARDS develops quickly after several conditions, traumas, or insults. However, it needs to be confirmed that the condition does not result from heart or renal failure or overhydration. Diffuse alveolar damage (DAD) is designated as a specific pathological finding for ARDS. For more than two decades, the definition set forth by the American-European Consensus Conference (AECC) has been used for the clinical diagnosis of ARDS [2], and a newer definition with better specificity has long been awaited. In 2011, a draft of a revised definition was presented at the 24th Annual Congress of the European Society of Intensive Care Medicine in Berlin, and its final version was published in *JAMA* in May 2012 [3]. In the revised Berlin definition, the term ARDS was redefined as a broader concept including a milder condition of lung injury; therefore, it became equivalent to acute lung injury (ALI), which was the previous AECC definition.

The revised ARDS definition was significantly improved by the inclusion of timing, underlying conditions, and the mandated determination of the PaO_2_/FIO_2_ ratio under positive airway pressure. However, no new parameters or biomarkers were adopted. In this review, the current understanding of the basic ARDS pathophysiology and associated candidate biomarkers will be discussed.

## Review

### Regulation of vascular permeability

The essential pathophysiology of ARDS includes increased pulmonary microvascular permeability. The process of water passage from the capillaries to the alveoli is presented with several physical barriers, including endothelial and epithelial cell layers, the basement membrane, and the extracellular matrix. Among these barriers, water passage (permeability) across endothelial and epithelial cell layers is actively regulated. Increased vascular permeability in ARDS is the result of several independent mechanisms. First, tissue injury and the resultant destruction of the pulmonary microvascular architecture contribute to a direct leak of blood components from the capillaries to the alveoli. In addition, endothelial and epithelial permeability is dynamically regulated by a set of inter- and intracellular molecules, the dysregulation of which may also induce increased vascular permeability. In order to protect the lungs from pulmonary edema, the pulmonary lymphatic system and epithelial water channels play important roles in pumping water out of extravascular space. However, when vascular leakage surpasses the capacity of these compensating systems, clinical pulmonary edema develops. There are multiple mechanisms by which vascular permeability is regulated. Sphingosine-1 phosphate (S1P) binds to its receptor, S1P1, and regulates vascular permeability through non-muscle myosin light chain kinase (nmMLCK) and the Rho family GTPase pathway [4]. In addition, angiopoietin-1 (Ang-1) binds to its receptor, tie-2, to stabilize the vasculature through the activation of Syx and Rho A [5]. In contrast, angiopoietin-2 (Ang-2) is produced by activated endothelial cells and competes with Ang1 for tie2 binding to destabilize vascular junctional formation [6]. The dysregulation of any of these mechanisms may lead to a change in vascular permeability; therefore, these factors may represent potential biomarkers for ARDS.

### The innate immune system and inflammation

Acute inflammation of and neutrophil accumulation in the lungs are commonly observed in both patients with ARDS and animal models of the disease. Extensive research has revealed the pathogenic roles of neutrophil-mediated acute inflammation in ARDS development [7]. Neutrophils release cytotoxic molecules, including granular enzymes, reactive oxygen metabolites, bioactive lipids, and cytokines, and induce the formation of neutrophil extracellular traps (NETs) [8]. In addition to causing tissue necrosis, these cytotoxic molecules induce apoptosis and autophagy, each of which causes tissue injury and cell death, which are characteristic of ARDS [9].

Numerous proinflammatory cytokines play major roles in acute inflammation and the development of inflammatory lung diseases, including ARDS. Among these, tumor necrosis factor alpha (TNFα) and interleukin 1beta (IL-1β) can induce ALI when administered to animals, and their levels are also elevated in the lungs of ARDS patients. Therefore, they are thought to be key pathogenic cytokines in ARDS. In addition, a neutrophil chemotactic chemokine, interleukin 8 (IL-8, CXCL8), is important because its neutralizing antibody was protective against the development of ALI in animal models, and IL-8 levels are elevated in the lungs of ARDS patients [10]. Additional cytokines and chemokines are involved in the development of ARDS, including IL-18 and IL-33, both of which, like IL-1β, are regulated by the inflammasome/caspase-1 pathway [11, 12]. These cytokines may represent good targets for antimediator therapy for ARDS as well as become potential biomarkers of ARDS.

Recently, pattern recognition receptors (PRRs) were demonstrated to play a key role in innate immunity [13]. PRRs are cell-surface or cytosolic proteins expressed by innate immune cells, and each is activated by a specific molecule(s). PRR ligands are divided into two categories, namely, pathogen-associated molecular patterns (PAMPs) and damage (danger)-associated molecular patterns (DAMPs). PAMPS are extrinsic molecules derived from various microorganisms, while DAMPs are intrinsic molecules derived from injured cells or extracellular molecules. When these PRRs are activated, nuclear factor (NF)-κB translocates to the nucleus, predominantly through a myeloid differentiation primary response gene 88 (MyD88)-dependent mechanism. Activation of PRRs also leads to the transcription of proinflammatory cytokines such as TNFα, IL-1β, and IL-8. Table 1 lists the major PRRs and their counterpart PAMPs and DAMPs.

**Table 1:** **Representative pattern recognition receptors** (**PRRs**) **and their ligands**

Family	Member	PAMPs	DAMPs
TLR	TLR1	Lipopeptides, lipoarabinomannan	Serum amyloid A protein
	TLR2	Lipopeptides, LTA, lipoarabinomannan, mannan, virus structural protein, zymosan, β-glucan	HMGB1, serum amyloid A protein
	TLR3	dsRNA	
	TLR4	LPS, virus structural protein	HMGB1, HSP60, HSP70, S100, HA, fatty acid
	TLR5	Flagellin	
	TLR6	Lipopeptides, zymosan, β-glucan	HA
	TLR7	ssRNA	
	TLR8	ssRNA	
	TLR9	CpG-DNA	Histone, mitochondrial DNA, self-DNA-containing immune complexes
NLR	NOD1	DAP-type PGN	
	NOD2	MDP	
	NLRC4	Flagellin, bacterial secretion systems	
	NLRP3	Pore-forming toxins, MDP	Nucleic acid, ATP, uric acid, HA, silica
RLR	RIG-I	dsRNA	Immunocomplex of snRNPs
Immunoglobulin superfamily	RAGE		AGEs, HMGB-1, S100B, transthyretin, amyloid-β peptide, Mac-1 integrin

*PRRs* pattern recognition receptors, *PAMPs* pathogen-associated molecular patterns, *DAMPs* damage (danger)-associated molecular patterns, *TLR* toll-like receptor, *LTA* lipoteichoic acid, *HMGB-1* high-mobility group box 1, *LPS* lipopolysaccharide, *HSP* heat shock protein, *HA* hyaluronic acid, *NLR* nucleotide-binding oligomerization domain (NOD)-like receptor, *DAP-type PGN* diaminopimelic acid containing peptidoglycan, *MDP* muramyl dipeptide, *NLRC4* NLR family CARD domain containing 4, *NLRP3* NLR family pyrin domain containing 3, *ATP* adenosine triphosphate, *RLR* retinoic acid-inducible gene-I (RIG-I)-like receptor, *RAGEs* receptor for advanced glycation end products, *AGEs* advanced glycation end products.

Infection, including severe sepsis and pneumonia, is the leading predisposing factor for ARDS. In this regard, the pathogenic roles of lipopolysaccharide (LPS) have been thoroughly examined. Because other PAMPs can induce proinflammatory reactions, it is reasonable to speculate that they also play important roles in the development and progression of ARDS. In addition, because tissue destruction (i.e., multiple trauma and burn injuries) is a major predisposing factor for ARDS, we can speculate that DAMPs play critical roles in its onset and/or progression. The high-mobility group box 1 protein (HMGB1) was one of the earliest discovered nuclear binding proteins demonstrated to function as a DAMP [14]. This protein not only leaks from damaged cells, but its production is also induced in activated dendritic cells and macrophages. HMGB1 can potently induce inflammation through its interaction with multiple receptors, including the receptor for advanced glycation end products (RAGE), toll-like receptor 2 (TLR2), and toll-like receptor 4 (TLR4). Initially, a pathogenic role of HMGB1 was reported in association with sepsis; subsequently, its involvement in ARDS was also revealed [15, 16]. Histone, another nuclear binding protein, is released into the circulation after trauma and can induce inflammation and ALI in animal models [17]. Further, mitochondrial DNA can induce the production of IL-8 and thus may play a role in ARDS as a DAMP [18]. At present, however, little is known of the pathogenic roles of PRRs, PAMPs, and DAMPs in ARDS, and their involvement needs to be clarified in future studies.

### Currently available biomarkers in clinical practice

Differentiating similar diseases or conditions from ARDS remains to be a matter of great importance. Currently, only a few biomarkers are clinically available for this purpose. For example, brain natriuretic peptide (BNP) is used for differentiation between ARDS and hydrostatic pulmonary edema, although its usefulness remains controversial [19, 20]. Procalcitonin is increased in bacterial infection, but not in viral or fungal infection; it may be useful for discriminating between bacterial pneumonia and ARDS. However, because the sensitivity of procalcitonin is as high as 70% for bacterial pneumonia and because bacterial pneumonia and sepsis are common predisposing conditions for ARDS, its utility is limited [21].

Predicting the severity of illness is also important to develop a specific diagnostic strategy for each patient with ARDS, but the PaO_2_/FIO_2_ ratio is the sole clinical parameter used for this purpose. The importance of biomarkers is underscored by the fact that they can also be utilized to predict response to therapy and prognosis. However, no ARDS-specific biomarkers are currently available for these purposes.

### Humoral factors as biomarkers of ARDS

As discussed above, various humoral factors have been identified as candidate biomarkers of ARDS (Table 2). Among the proinflammatory cytokines, TNFα, IL-1β, interleukin 6 (IL-6), and IL-8 are elevated in the bronchoalveolar lavage fluid (BALF) of ARDS patients, and their levels were reportedly higher in non-survivors than in survivors [22]. We previously showed that IL-8 levels in BALF were higher in patients with ARDS and inhalation injury [10, 23]. These levels were also able to predict the degree of lung oxygenation impairment in inhalation injury. Recent secondary analysis of the ARDS Clinical Network's (ARDSnet) activated protein C study, where various candidate biomarkers of ARDS were assessed, showed that plasma plasminogen activator inhibitor 1 (PAI-1) and IL-6 were correlated with the oxygenation index (mean airway pressure × FIO_2_/PaO_2_). Furthermore, ventilator-free days were significantly shorter in patients with higher levels of IL-6, IL-8, and thrombomodulin, which were associated with poor patient outcomes [24]. Among these three molecules, the usefulness of IL-8 in predicting the outcome of ARDS was confirmed by several additional studies [25, 26]. A recent report from Harvard demonstrated that IL-18 is a new ARDS biomarker [12]. This study was independently performed by three affiliated hospitals and showed a consistent increase in plasma IL-18 levels in ARDS patients, while mortality was increased in direct proportion to plasma IL-18 levels.

**Table 2:** **Biomarkers of ARDS**

Name	Change in ARDS	Clinical prediction
Humoral mediators		
Cytokines, growth factors		
TNFα	BALF↑	Poor outcome
IL-1β	BALF↑	Poor outcome
IL-2	Blood↑	Development
IL-4	Blood↑	Development
IL-6	Blood↑, BALF↑	Poor outcome
IL-8	Blood↑, BALF↑	Development and severity (BALF), poor outcome
IL-18	Blood↑	Poor outcome
VEGF	ELF↑	Better outcome
KGF	BALF↑	Poor outcome
GDF-15	Blood↑	Poor outcome
Ang-2	Blood↑	Development, poor outcome
Neutrophil elastase	Blood↑	Development and severity
Leptin	BALF↑	Poor outcome
Coagulation/fibrinolysis factors		
PAI-1	Blood↑	Poor outcome
Thrombomodulin	Blood↑	Poor outcome
von Willebrand factor	Blood↑	Development
Protein C	Blood↓	Poor outcome
Substances released from injured or activated tissues		
DAMPs		
HMGB-1	Blood↑	Poor outcome
DNA	BALF↑	Poor outcome
Endothelial cells		
Soluble P-selectin	Blood↑	Poor outcome
Soluble ICAM-1	Blood↑	Poor outcome
Epithelial cells		
Soluble RAGE	Blood↑	Poor outcome
SP-B	Blood↑	Development
SP-D	Blood↑	Poor outcome
CC-16	Blood↑	Poor outcome
Laminin γ2	ELF↑	Poor outcome
KL-6	Blood↑, BALF↑	Poor outcome

*BALF* bronchoalveolar lavage fluid, *ELF* epithelial lining fluid, *TNFα* tumor necrosis factor alpha, *IL* interleukin, *VEGF* vascular endothelial growth factor, *KGF* keratinocyte growth factor, *GDF-15* growth differentiation factor-15, *Ang-2* angiopoietin-2, *PAI-1* plasminogen activator inhibitor 1, *DAMPs* damage (danger)-associated molecular patterns, *HMGB-1* high-mobility group box 1, *ICAM-1* intercellular adhesion molecule 1, *RAGE* receptor for advanced glycation end products, *SP* surfactant protein, *CC-16* Clara cell specific protein 16, *KL-6* Krebs von den Lungen-6.

Several growth factors have been determined to be candidate biomarkers of ARDS. In this regard, the lung levels of vascular endothelial growth factor (VEGF) and keratinocyte growth factor (KGF) were shown to correlate with the severity of illness and reflect patient outcome [27, 28]. Furthermore, secondary analysis of the ARDSnet's Fluid and Catheter Treatment (FACT) study revealed that plasma levels of growth differentiation factor-15 (GDF-15) were increased in proportion to 60-day mortality [29]. Another recent study showed that Ang-2, a competitor of Ang-1 and a regulator of vascular permeability (as mentioned earlier), could predict the prognosis of ARDS [30].

As described, among inflammatory cells, neutrophils play dominant roles in inducing ARDS through the release of various cytotoxic substances and mediators, including granular enzymes, reactive oxygen species, bioactive lipids, cytokines, and NETs. Therefore, these neutrophil-derived molecules can be candidate biomarkers of ARDS. Neutrophil elastase, a major granular enzyme with potent non-specific tissue destruction activity, forms a complex with alpha 1-antitrypsin (NE-AT) soon after release from activated neutrophils. We have previously shown that the levels of the NE-AT complex were increased in ARDS patients and were significantly higher in a subgroup of patients with clinical deterioration after admission than in a subgroup without deterioration [31].

Leptin, a hormone involved in the regulation of energy intake and expenditure, was also shown to contribute to ARDS development. Epidemiological data demonstrated the low incidence of ARDS among patients with diabetes mellitus; however, the reason for this is unknown [32, 33]. Recently, a decrease in leptin levels in these patients was shown as a potential key mechanism underlying this epidemiological finding. In an animal experiment, leptin induced the expression of transforming growth factor beta (TGF-β) and the production of collagen types I and II in the presence of TGF-β, and leptin-deficient mice were resistant to the development of ALI [34]. Furthermore, in non-obese patients with ARDS, leptin levels in BALF correlated with TGF-β levels. The duration of artificial ventilation and ICU stay was significantly longer in a subgroup of ARDS patients with higher leptin levels in BALF than in those with lower leptin levels in BALF [34]. These results suggest that leptin can be a candidate biomarker of ARDS.

### Substances derived from activated cells or injured tissues as biomarkers of ARDS

Substances derived from activated cells or injured tissues can also reflect the degree of inflammation or tissue injury and, consequently, the severity of ARDS. In addition to the earlier discussed pathogenic role of HMGB1 in ARDS, it was shown to be a candidate biomarker of ARDS, along with soluble RAGE [15]. Excessive formation and ineffective clearance of neutrophil extracellular trap in alveolar space would be responsible for the pathogenesis of ARDS. The increase in DNA decorated with proteases and histone in BALF was observed in cystic fibrosis [35] and acute inhalation injuries [36]. Thus, DNA in BALF could also become the candidate as biomarker for ARDS. Similarly, histone may be useful as an ARDS biomarker in patients with lungs subjected to multiple trauma [17]. As the roles of DAMPs in the pathophysiology of ARDS are revealed, their utility as biomarkers will also be clarified.

Among endothelial cell-derived molecules, plasma levels of soluble P-selectin and soluble intercellular adhesion molecule (sICAM-1) were reported as candidate biomarkers. The potential of sICAM-1 was demonstrated by multicenter studies [37, 38]. Additional epithelial cell-derived molecules that represent candidate ARDS biomarkers include sialylated carbohydrate antigen Krebs von den Lungen-6 (KL-6, a fragment of MUC1 mucin), surfactant protein B (SP-B) [39], surfactant protein D (SP-D) [25, 26, 40], Clara cell protein CC-16 [41], and the gamma-2 chain of laminin-5 (an extracellular matrix protein with cell adhesive properties) [42].

In 2014, an article that focused on a new meta-analysis of plasma biomarkers for ARDS was published [43]. The authors analyzed 54 studies and found that KL-6, lactate dehydrogenase, soluble RAGE, and von Willebrand factor are strongly associated with ARDS diagnosis in the at-risk population. For outcome prediction, they found that IL-4, IL-2, Ang-2, and KL-6 were most strongly associated with mortality from ARDS.

## Conclusions

In parallel with progress in the understanding of ARDS pathophysiology, several molecules have been shown to be candidate biomarkers of this disease, with the clinical usefulness of some being confirmed by large-scale or multicenter studies. However, none of these candidates have been clinically applied for diagnosis or prediction of disease severity, response to therapy, and prognosis in patients with ARDS. Future studies, along with a search for new biomarker candidates, need to determine the potential application(s) of each candidate discussed here. This will lead to improved diagnosis and treatment strategies for patients with ARDS.
